# The Counter-Regulatory Renin-Angiotensin System: A Surprising Ally in the Field of COVID-19

**DOI:** 10.2174/0118715265352715250717101135

**Published:** 2025-07-30

**Authors:** Mariali Palacios-Cruz, Jairo Castellar-López, Juan Manuel Pretelt, Aileen Y. Chang, Evelyn Mendoza-Torres

**Affiliations:** 1 Grupo de Investigación Avanzada en Biomedicina, Faculty of Health, Exact and Natural Sciences, Universidad Libre Seccional Barranquilla, Barranquilla, Colombia;; 2 Internal Medicine Program, Faculty of Health, Exact and Natural Sciences, Universidad Libre de Colombia, Seccional Barranquilla, Barranquilla, Colombia;; 3 Department of Medicine, School of Medicine and Health Sciences, The George Washington University, Washington, District of Columbia, DC 20052, USA

**Keywords:** COVID-19, renin-angiotensin-aldosterone system, angiotensin conversion enzyme type 2, angiotensin (1-7), angiotensin (1-9), SARS- CoV-2, renin-angiotensin system

## Abstract

**Introduction:**

Over the past four years, SARS-CoV-2 and COVID-19 have become global health crises, spurring extensive research on virus behavior, complications, and treatments. The virus interacts with a component of the renin-angiotensin system (RAS), altering inflammatory, hypertrophic, and hemodynamic responses *via* binding to ACE2 found in organs like the heart, lungs, and kidneys.

**Objective:**

This review explores the RAS-COVID-19 interplay, focusing on key molecules like ACE2, Ang-(1-7), and Ang-(1-9), influencing susceptibility, severity, and treatments. It seeks to clarify ACE2's dual role in viral entry and protection and assess the therapeutic potential of balancing Ang-(1-7) and Ang-(1-9) to prevent disease progression and related complications.

**Methods:**

Studies were chosen through a systematic search in databases, such as PubMed, Scopus, and Web of Science. The inclusion criteria were centered on peer-reviewed research that explored the relationship between SARS-CoV-2 and important RAS molecules, including ACE2, Ang-(1–7), and Ang-(1-9), seeking information on therapies, severity, and susceptibility. Non-peer-reviewed articles and those lacking focus on RAS-COVID-19 interplay were excluded. Titles and abstracts were screened, followed by full-text assessment and data extraction for analysis.

**Results:**

Some studies indicate that the peptides Ang-(1-7) and Ang-(1-9) could provide protective effects against heart-related complications by counteracting the harmful impacts of the angiotensin II pathway, which is often exacerbated by SARS-CoV-2. Ang-(1-7) and Ang-(1-9) are recognized for promoting vasodilation, reducing inflammation, and preventing fibrosis, which can mitigate the heart damage typically associated with COVID-19.

**Discussion:**

ACE2, a component of the non-canonical RAS, is closely linked to SARS-CoV-2 and plays a pivotal role in the pathophysiology of COVID-19. Ang-(1-9) and Ang-(1-7) are produced by ACE2 and have demonstrated positive cardiovascular effects. In the context of COVID-19, Ang-(1-7) has shown protective effects in preclinical studies and clinical trials; however, more evidence is needed to support this effect.

**Conclusion:**

Further research, including clinical trials, is vital to understand and develop precise therapies for COVID-19 and similar infectious diseases.

## 1 INTRODUCTION

The emergence of the severe acute respiratory syndrome coronavirus 2 (SARS-CoV-2), causing coronavirus disease 2019 (COVID-19), has ignited extensive research in various fields, presenting ongoing challenges in virus isolation, detection, prevention, and vaccine development [1, 2]. In recent years, the people of the world have suffered from various non-infectious diseases such as diabetes, hypertension and infectious diseases such as COVID-19. These conditions have had a significant impact on public health, emerging as major challenges due to their widespread prevalence and the variability of their effects on individuals. As a result, they have contributed to a steady rise in global morbidity and mortality, placing increasing pressure on healthcare systems worldwide [3]. Understanding the biological processes that influence the host immune response to this disease has become crucial to combating its impact. COVID-19 significantly contributes to respiratory tract infections in humans, influenced by individual factors [4, 5]. Its high transmissibility (basic reproductive number (R0) of 2.2, which signifies that, on average, each infected person can potentially infect 2.2 others within a susceptible population) relies on the interaction between the virus's spike protein and the angiotensin-converting enzyme 2 (ACE2) receptor [6-8]. Consequently, the renin-angiotensin system (RAS) has become a focal point, particularly the ACE2 receptor, a cell surface carboxypeptidase and negative RAS regulator [6, 9]. Angiotensin production involves classical and counter-regulatory RAS pathways [10]. In the classical RAS, angiotensinogen is converted into Ang I, which angiotensin-converting enzyme (ACE) further transforms into Ang II, binding to angiotensin II type 1 (AT1R) and type 2 (AT2R) receptors [9]. Conversely, the counter-regulatory RAS features ACE2, converting Ang II into Angiotensin-(1-7) [Ang-(1-7)] or Ang I into Angiotensin-(1-9) (Ang-(1-9)) [9]. ACE2 primarily acts on Ang II due to its higher affinity [8]. Ang-(1-7) binds to the Mas receptor (MasR), inducing vasodilation, lowering blood pressure, and counteracting ACE-Ang II effects [8, 10, 11]. Due to the role of ACE2 as SARS-CoV-2 receptor and its contribution to Ang-(1-9) and Ang-(1-7) production, we explore peptide levels and their counter-regulatory functions during COVID-19 infection. Despite limited information, this article delves into the topic, conducting a comprehensive review of scientific literature and recent research. Our objective is to understand how molecular interactions within the RAS, both classical and counter-regulatory, influence the course of COVID-19, potentially paving the way for more effective and personalized therapies.

## 2 METHODOLOGY

To conduct this mini-review, we implemented a structured and rigorous literature search aimed at exploring the role of the counter-regulatory RAS in the pathophysiology of COVID-19. The literature search included multiple reputable biomedical databases, such as PubMed/MEDLINE, Scopus, Web of Science, and ScienceDirect. Our search utilized a combination of Medical Subject Headings and free-text terms, including “renin-angiotensin system”, “ACE2”, “angiotensin-(1-7)”, “Mas receptor”, and “COVID-19”. Boolean operators (AND, OR) were applied to refine and optimize the search strategy for sensitivity and specificity.

The inclusion criteria were defined to prioritize scientific rigor and relevance. We included peer-reviewed original research articles, systematic reviews, and meta-analyses published in English that addressed the counter-regulatory axis of the RAS, particularly ACE2, Ang-(1-7), and the Mas receptor in the context of SARS-CoV-2 infection and disease progression. Studies based on *in vitro*, *in vivo*, and clinical models were considered. Exclusion criteria involved preprints not yet peer-reviewed, articles unrelated to the counter-regulatory RAS axis, and publications with methodological flaws or lacking substantial evidence.

All articles were independently screened by the authors to minimize selection bias. Eligible studies were critically evaluated for methodological quality, relevance, and scientific contribution. The selected literature was then synthesized in a narrative format to highlight consistent findings, theoretical models, and experimental data that support the involvement of the counter-regulatory RAS in modulating immune and inflammatory responses during COVID-19. This approach ensures the transparency and reproducibility of our review and provides a scientifically robust foundation for the discussion and conclusions presented.

To further strengthen the quality of this review, we prioritized articles published in high-impact journals and those widely cited within the scientific community, ensuring the inclusion of influential and validated data. Discrepancies in article selection were resolved through discussion among the authors until consensus was reached. Additionally, the reference lists of key articles were manually screened to identify any relevant studies not captured during the initial database search, thus enhancing the comprehensiveness of the review process.

The selection and synthesis of the literature followed the principles of thematic analysis, focusing on the physiological functions of the counter-regulatory RAS components, their modulation during SARS-CoV-2 infection, and the potential clinical implications of their dysregulation.

## 3 COVID-19

Since its identification as the causative agent of COVID-19, extensive research has significantly improved our understanding of SARS-CoV-2. It has unveiled crucial insights into its characteristics, behavior, and heightened transmissibility, leading to a global pandemic with 767, 518, 723 cumulative cases and 6, 947, 192 deaths worldwide as of June 2023 [12-14]. SARS-CoV-2, which belongs to the Beta coronavirus genus within the Coronaviridae family, possesses a single-stranded, positive-sense RNA genome. It features a diameter of 60 to 140 nm and distinctive spikes measuring 9 to 12 nm, giving them a corona-like appearance. This virus contains the spike protein (S), which is essential for interacting with the ACE2 surface receptor on host cells and initiating infection *via* the S1 and S2 subunits [15-18]. These subunits, together with the transmembrane protease serine protease-2 (TMPRSS-2), are critical for facilitating viral and host membrane binding, promoting internalization by endocytosis, followed by replication and translation, Fig. (**1**) [18].

CoV's structural proteins (S, N, M, and E) play a crucial role in facilitating host cell entry. The hemagglutinin esterase (HE) protein enhances the S protein's binding activity and facilitates viral spread across mucosal surfaces [19-21]. SARS-CoV-2 specifically targets the ACE2 receptor for cell entry, resulting in ACE2 receptor downregulation and increased angiotensin-2 production [19-21]. This process can potentially cause lung injury by affecting multiple organs due to the widespread distribution of ACE2 receptors. Immune injury results from excessive T-cell activation, and alveolar macrophages and epithelial cells [19-21] initiate lung inflammation. The clinical course of infection includes viremia, acute pneumonia, and recovery stages, with an incubation period from 1 to 14 days, potentially extending up to 24 days [19-21]. Common symptoms include fever, cough, and difficulty breathing, along with weakness, fatigue, nausea, vomiting, diarrhea, and alterations in taste and smell perception [22]. Imaging techniques reveal lung abnormalities like ground-glass opacification, accompanied by organ-specific irregularities [21].

## 4 CLASSICAL RENIN-ANGIOTENSIN SYSTEM (RAS)

**Fig. (1):**
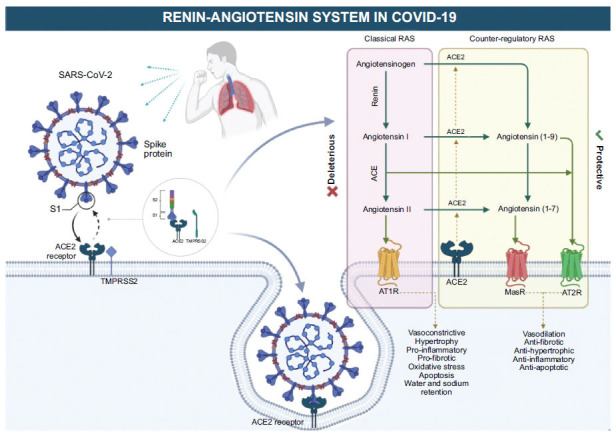
Interaction of SARS-CoV-2 with the renin-angiotensin system in COVID-19 and its antagonistic effects on cardiovascular function.

The classical RAS, comprising renin and angiotensinogen, involves the sequential transformation of angiotensinogen into Ang I and Ang II peptides through ACE, which act as the main biologically active mediators of RAS, exerting their effects *via* specific receptors known as angiotensin type 1 receptor (AT1R) and angiotensin type 2 receptor (AT2R) [23, 24]. The classical RAS has a crucial role in diseases involving inflammatory, hypertrophic, fibrotic, and systemic vasoconstriction processes [23, 25]. Consequently, understanding the interplay and antagonism between the classical and counter-regulatory RAS is very important for understanding transmission and severity of COVID-19 [23-25]. Essentially, the classical RAS is associated with vasoconstriction, sodium retention, and elevated blood pressure, potentially contributing to inflammation and organ damage during a COVID-19 infection [26] (Fig. **1**). These effects are due to the interaction of angiotensin II with AT1R, which are opposite to the effects that result from the interaction with AT2R [27].

## 5 COUNTER-REGULATORY RAS

In contrast to the classical RAS, the counter-regulatory RAS includes ACE2, neprilysin (NEP), almandine, proto-oncogene Mas receptor (MasR), Mas-related G protein-coupled receptor member D, Ang-(1-9), and Ang-(1-7), originating from Ang I and Ang II conversions by specific enzymes [28-30]. ACE2 and NEP enzymes convert Ang II into Ang-(1-7), and ACE2 also transforms Ang I into Ang-(1-9) while processing Angiotensin A, a structural analog of Angiotensin II, into almandine [29, 30]. ACE2, a glycoprotein primarily expressed in vascular endothelial cells and renal tubular epithelium, serves as the primary receptor for SARS-CoV-2. In contrast to ACE, ACE2 functions as a carboxypeptidase, and exists in two isoforms: one anchored to cell membranes (mACE2) and the other soluble (sACE2). These isoforms facilitate the virus's entry into host cells [31-33]. ACE2 also plays a vital role in producing Angiotensin-(1-7), which is found in essential organs like the heart, brain, and kidneys. This heptapeptide delivers various beneficial effects *via* MasR (Mas receptor) at the vascular level, including vasodilation, vascular protection, fibrosis prevention, inhibition of cell proliferation, and anti-inflammatory properties, which collectively enhance cardiac and pulmonary functions [34]. Although less explored, Ang-(1-9) holds significance within the RAS. It competes with Ang II for active site of ACE, leading to increased levels of Ang-(1-7) and stimulating bradykinin release in endothelial cells [35]. As a result, it enhances the production of nitric oxide and encourages a urinary natriuretic response, which generates vasodilation and contributes to the reduction of blood pressure [36]. The intricate relationship between ACE2, Ang-(1-7), and Ang-(1-9) levels in COVID-19 patients has multifaceted effects on disease severity, symptoms, and susceptibility, highlighting their clinical significance. However, the variety of findings among different study groups emphasize the need for further research to comprehensively understand their clinical implications and therapeutic potential in COVID-19.

## 6 ACE2 AND COVID-19

Understanding the association between ACE2 and COVID-19 is vital for understanding and combating SARS-CoV-2 infections. ACE2 expression changes during infection, impacting various organs, especially in the elderly, such as the heart, respiratory system, endothelial cells, and vascular smooth muscle cells, regulating the local RAS without altering blood pressure [37-39]. ACE2 converts Ang II to Ang-(1-7), a vasodilator with cardioprotective effects present in multiple organs and notably higher in male plasma, potentially indicating male-genitourinary COVID-19 risk [40, 41]. SARS-CoV-2 has implications for microvasculature, leading to endotheliitis and inflammation [42]. Recent studies indicate elevated ACE2 plasma concentrations in individuals with COVID-19 compared to those who are unaffected. Furthermore, the seriousness of a SARS-CoV-2 infection varies based on the COVID-19 variant and ACE2 expression levels. The consequences of these alterations are further influenced by individual-specific factors like gender, age, body mass index (BMI), smoking behavior, and the presence of comorbidities [43, 44]. Numerous studies have examined the complex link between ACE2 and COVID-19, shedding light on its potential impact on disease susceptibility, severity, and therapeutic interventions. Maza *et al*. 2022 [45] conducted an observational study, revealing a strong link between higher serum ACE2 levels and reduced infection risk. This suggests that ACE2 might act as a protective factor, influencing SARS-CoV-2 virus entry and replication. Similarly, an observational study by Wallentin *et al*. 2020 provided valuable insights. Elevated ACE2 levels were associated with clinical indicators and biomarkers linked to aging, cardiovascular disease, and diabetes. This suggests that patients with these comorbidities might have increased ACE2 expression, potentially making them more susceptible to COVID-19 or experiencing more severe outcomes [45, 46]. In 2022, Elrayess *et al*. [47] explored hypertensive COVID-19 patients in a retrospective, finding that elevated ACE2 levels correlated with disease severity alongside lower Ang II levels. This suggests the potential role of the ACE2/Ang II balance in COVID-19 in individuals with hypertension. However, Files *et al.* [47, 48] (2021) highlighted discrepancies between circulating and local RAS levels in COVID-19, emphasizing the importance of considering local tissue changes in understanding the disease [49]. The primary cell types that express ACE2, which acts as SARS-CoV-2 receptor, include adipocytes, thyroid, heart, small intestine, testes, kidneys, and epithelial cells found in the apical pole of the lungs [50]. Age has been identified as a risk factor for fatal complications, particularly in older age groups when Ang II and AT1R levels are higher, and MasR is lower. Chronic degenerative disorders like obesity, diabetes mellitus, hypertension, and cardiovascular disease are also more common in older adults [50, 51]. The degree of pulmonary involvement ranges from mild pneumonia to severe hypoxia linked to respiratory failure. SARS-CoV-2 causes interstitial inflammation and alveolar injury in the lungs, which raises proinflammatory cytokine and chemokine levels. Additionally, bleeding, vascular edema, thromboembolism, and hyaline thrombosis of the pulmonary vessels all involve the microvasculature. Pulmonary infarction, bleeding, pulmonary hypertension, and secondary ventricular stress are all brought on by this microthrombus development [52]. Furthermore, fibrotic abnormalities and persistent continuous dyspnea may be evident in patients with severe cases of pneumonia [53]. By generating inflammation and a prothrombotic environment, SARS-CoV-2 injures endothelial cells. Endothelial injury from various vascular bed infections in the liver, kidneys, heart, small intestine, and lungs can lead to microvascular dysfunction and excessive thrombin generation. It can also start complement pathways that produce thrombo-inflammation. This might result in the pulmonary circulation's microvascular and macrovascular thrombosis [54]. More than 7.5% of cardiac cells express ACE2, which promotes viral entrance into cardiomyocytes and results in direct cardiotoxicity at the cardiovascular level. Acute coronary syndrome, cardiomyopathy, arrhythmias like atrial fibrillation, heart block, ventricular arrhythmias, and cardiogenic shock from heart failure can all be caused by vascular inflammation mediated by infection. It can also result in myocardial inflammation, a state of hypercoagulability, and direct inhibitory myocardial suppression. Hypoxemia and vasoconstriction can potentially lead to the development of persistent myocardial ischemia [54]. Studies reveal that acute renal failure is caused by the virus-induced cytopathic impact, which manifests as hematuria, proteinuria, hyperkalemia, and acidosis. The incidence of acute kidney injury rose following this pandemic. Furthermore, research on individuals with severe COVID-19 has revealed a pattern of hepatic damage, including modest lobular and portal inflammation, moderate microvesicular steatosis, and increased liver enzymes that may be brought on by the infection or medications used to treat it [55]. Lastly, there is a greater chance of progression to a severe stage in people who have both obesity and diabetes mellitus. Additionally, it has been shown that COVID-19 results in anomalies related to the metabolism of glucose, exacerbating hyperglycemia and typical diabetic ketoacidosis and making patient treatment even more difficult. Obesity is thought to be an inflammatory condition at the cellular level, with changes in cytokines, chemokines, and adipokines, all of which exacerbate COVID-19 problems. Acute ischemic stroke and other neurological symptoms are caused by the pathophysiology of SARS-CoV-2, which induces cytokine storm and damages the blood-brain barrier and cerebral vasculature [56]. Thus, the regulation of ACE2 expression and its susceptibility to COVID-19 in each organism is influenced by various underlying conditions and comorbidities. Also, by altering the physiological ACE/ACE2 balance, SARS-CoV-2 is observed to intervene by activating the Ang II/AT1R pathways and leading to significant complications in the disease [18]. In the scope of future research, consideration of ACE2 as a therapeutic target to mitigate the spread of the virus in the context of COVID-19 is proposed. This approach includes administration of the soluble form of ACE2 (sACE2) and inhibition of the interaction between SARS-CoV-2 and hACE2. [57, 58] Some condition like the Inflammatory conditions like inflammatory bowel disease (IBD) may influence COVID-19 outcomes through their interaction with RAS, particularly *via* the ACE2 receptor, which is used by SARS-CoV-2 to enter host cells. Since ACE2 is expressed in both lung and intestinal tissues, this may explain the respiratory and gastrointestinal symptoms observed in infected patients. While some studies suggest that IBD increases ACE2 expression in the gastrointestinal tract, others dispute this claim; however, ulcerative colitis (UC), a subtype of IBD, research found a relationship with IBD with a higher ACE2 expression and an increased risk of severe COVID-19 outcomes, including hospitalization and mortality. Beyond ACE2 expression, the use of immunosuppressive medications in IBD treatment further complicates this relationship, as patients on corticosteroids or 5-aminosalicylic acid (5-ASA) exhibit higher rates of hospitalization, Intensive Care Unit (ICU) admission, and death, suggesting that severe inflammation and immune dysregulation, rather than IBD itself, may drive worse outcomes. Understanding how inflammatory diseases interact with the RAS pathway could provide insights into broader mechanisms by which underlying conditions influence viral infections, and future research should explore whether similar interactions exist in other inflammatory diseases, such as rheumatoid arthritis or chronic kidney disease, to improve risk stratification and treatment strategies [59]. Understanding the most up-to-date and standardized methods for the diagnosis of COVID-19 and its relationship with RAS is fundamental. However, it is also essential to analyze how individual patient conditions modify the response to treatments and preventive methods against the virus. Various diseases, such as solid tumors, hematological malignancies, autoimmune diseases, IBD, transplantation and hemodialysis, influence the risk of infection and the body's ability to counteract it, according to studies such as that of Cho *et al*. (2022) [60]. RAS plays a key role in the severity of COVID-19, as SARS-CoV-2 uses ACE2 as a gateway receptor, reducing its expression and generating an inflammatory imbalance. Patients with obesity, diabetes, hypertension, autoimmune diseases, cancer or immunosuppression present an altered regulation of RAS, exacerbating inflammation and vascular damage. In addition, immunosuppressive and RAS-modulating treatments, such as ACEIs, ARBs (Angiotensin II receptor blockers) or JAK (Janus kinase) inhibitors, affect immune response and vaccination efficacy. Optimizing RAS modulation by AT1R blockers or MasR agonists could improve immunity and reduce the severity of COVID-19 in these patients. This perspective underscores the need for personalized therapeutic strategies that consider both the pathogenesis of the virus and the underlying conditions of each patient [60]. In summary, these studies offer valuable insights into ACE2 and related biomarkers in COVID-19. However, further research is crucial to fully comprehend this intricate relationship and to develop targeted therapies.

## 7 ANG-(1-7) AND COVID-19

During the COVID-19 pandemic, significant interest was generated in Ang-(1-7) due to its involvement in SARS-CoV-2 infection. An association between lower levels of Ang-(1-7) and severity of COVID-19 disease was observed, as demonstrated by investigations such as that of Carpenter *et al*. in 2022, which revealed a progressive decrease in Ang-(1-7) levels in relation to symptom duration, especially in hospitalized and critically ill patients [61]. However, other studies, such as the one conducted by Rostamzadeh *et al*. in 2023, showed that these levels may also be influenced by the presence of risk factors, such as arterial hypertension (AHT). In situations in which patients present with both COVID-19 and AHT, a decrease in Ang-(1-7) levels has been observed. Therefore, it has been suggested that treatment with Ang-(1-7) could be beneficial in mitigating the cardiopulmonary level effects of SARS-CoV-2, due to its antihypertensive, anti-inflammatory, antithrombotic, antiarrhythmic, and vasodilatory properties [62]. Furthermore, viral infections are known to trigger cellular inflammation and oxidative stress [41]. In the case of SARS-CoV-2, it has been observed that a higher ratio of Ang II to Ang-(1-7) may lead to an increase in the expression of AT1R and ACE while reducing the levels of MasR and AT2R [62]. This overall imbalance could potentially exacerbate the pathologies associated with COVID-19. Interestingly, this scenario might be particularly relevant in males with COVID-19, as they generally exhibit a lower ACE2-Ang-(1-7) tone compared to females, which might limit their ability to buffer an activated ACE-Ang II-AT1R axis, potentially contributing to more severe outcomes in male patients [62]. The findings about angiotensin, such as Ang-(1-7), suggest their potential as therapeutic agents for COVID-19 treatment. In another study by Mohammad *et al*. (2022), the relationship between Ang-(1-7) levels and COVID-19 severity was explored and they found that higher levels of Ang-(1-7) were associated with decreased disease severity, suggesting that synthetic Ang-(1-7) may hold therapeutic promise in treating and reducing complications of COVID-19 [34]. The potential therapeutic of synthetic Ang-(1–7) in the treatment of COVID-19 opens new possibilities for drug development [33]. By supplementing the body with exogenous Ang-(1-7), it may be possible to enhance the natural protective mechanisms against the virus and mitigate its harmful effects on the cardiovascular and respiratory systems [34]. However, it is essential to approach this potential therapy with caution and further investigate its safety and efficacy through rigorous clinical trials. For example, Wagener *et al*. (2022) in their randomized, placebo-controlled, double-blinded pilot study, demonstrated that a form of Ang-(1–7), TXA-127, was safe to administer in patients with severe COVID-19 infection, suggesting its potential as a therapeutic option for these cases [63, 64]. Ang-(1-7) has also emerged as a key player in mitigating pulmonary fibrosis, myofibroblast generation, and collagen synthesis by modulating pathways involved in Ang II-mediated tissue fibrosis in COVID-19 [64]. Notably, Ang-(1-7) exerts inhibitory effects on phosphorylation, downregulates pro-fibrotic activity, and counters endothelial dysfunction and cellular differentiation by thwarting NADPH oxidase-induced oxidative stress. These multifaceted actions underscore the promising therapeutic potential of Ang-(1-7) in managing fibrosis and COVID-19 patients, with Phase I ensuring safety and Phase II testing efficacy against placebo, including outcomes like oxygen-free days, hospital stay, ICU days, mortality, and ventilation inflammation across diverse conditions, including those linked to Ang II-mediated tissue fibrosis [65]. ACE2 provides protection in cases of acute lung injury. The ACE2/ANG-(1-7) axis can reduce tissue damage caused by viruses by preventing alveolar cell apoptosis and reducing endothelial cell activation, loss of barrier function, and edema [66]. By reducing pulmonary hypertension, pulmonary fibrosis, and arterial remodeling, ACE2 reduces lung injury and vascular damage in experimental animals [67]. Since Ang-(1-7) has several beneficial effects on the cardiovascular system, including vasodilation, myocardial protection, antiarrhythmic, antihypertensive, and positive inotropic effects, ACE2 overexpression and Ang-(1-7) action ameliorate mi-induced cardiac remodeling at the cardiovascular level [68]. Ang-(1-7) administration in normotensive animals causes vasodilatation *via* arterial baroreceptor discharge, which raises cardiac output and lowers peripheral resistance to keep blood pressure from fluctuating. Furthermore, Ang-(1-7) has antithrombotic effects in mice *via* raising nitric oxide and platelet prostacyclin levels [69]. Additionally, ACE2, expressed in a variety of cell types, inhibits Ang-II activity, and stimulates the Ang-(1-7)/MasR axis in cardiomyocytes, cardiac fibroblasts, coronary endothelial cells, epicardial adipose tissue, and coronary vascular endothelium [69, 70]. ACE2 has been identified as having a vital involvement in heart failure, pulmonary and systemic hypertension, myocardial infarction, and diabetes in animal models of human illness using both loss-of-function and gain-of-function techniques [70]. The production of Ang-(1-7), which has been proven to suppress proinflammatory cytokines including TNF-α (Tumor Necrosis Factor-α) and IL-6, is responsible for many of the effects of ACE2 that have been observed [71]. Through the stimulation of pancreatic beta cells, Ang-(1-7) has also been demonstrated in other mouse experiments to improve insulin secretion [72]. Research conducted on cells has demonstrated that it also lessens the amount of reactive oxygen species, which lessens damage to cells. An investigation conducted on obese people revealed that Ang-(1-7) can decrease hemodynamic abnormalities [73]. Ang-(1-7) is well tolerated and does not appear to be harmful [48]. The worldwide health crises caused by COVID-19 during the last four years have sparked a great deal of study on viral behavior, consequences, and therapies [74]. The virus binds to ACE2, which is present in organs including the heart, lungs, and kidneys, and then interacts with the renin-angiotensin-aldosterone system (RAS) to modify hemodynamic, hypertrophic, and inflammatory responses [75]. Although elevated ACE2 may offer protection against SARS-CoV-2, its effect on the severity of the illness is yet unknown [70]. The RAS-COVID-19 relationship is examined in this review, with particular attention to important molecules that affect susceptibility, severity, and therapies, such as ACE2, Ang-(1-7), and Ang-(1-9) [76]. While some research points to protection, other studies stress the necessity of balancing these chemicals to stop the spread of illness. Completing clinical trials and other research is essential to comprehending COVID-19 and creating targeted treatments [77]. The role of Ang-(1-7) in COVID-19 is intriguing but requires more research to unlock its full therapeutic potential. Understanding this complex system could enhance COVID-19 management and treatment. However, more studies are needed to fully comprehend the complexities of this intricate regulatory system and its implications for clinical practice.

Understanding the role of pathophysiology in COVID-19 detection is crucial for accurate and timely diagnosis, as diagnostic strategies have evolved alongside advances in viral genetics. While PCR remains the standard, its sensitivity can be affected by viral load fluctuations and mutations, prompting the use of imaging techniques such as chest radiography, lung ultrasound, magnetic resonance imaging (MRI), positron emission tomography (PET), and Computed Tomography (CT), though their effectiveness varies. To enhance detection, Ali *et al.* (2024) [78] propose a machine learning model combining 16 deep learning architectures (*e.g*., EfficientNet-b0, ResNet-50, DarkNet-53) with 10 optimization techniques (*e.g*., MRFO, MPA), achieving a 97% accuracy rate. These advancements not only improve COVID-19 diagnostics but also provide insights into the virus’s interaction with the RAS, which contributes to systemic inflammation and lung dysfunction. By identifying patterns like ground-glass opacities and pulmonary fibrosis, deep learning models can differentiate COVID-19 from other infections while indirectly assessing RAS activity, paving the way for more integrated and personalized diagnostic strategies [78].

Modern advancements in deep learning and robotics are transforming patient detection and classification, particularly in the context of COVID-19, where disease severity and presentation vary widely, exacerbating staffing shortages and highlighting the need for innovative healthcare solutions. To address this challenge, an ensemble deep learning model integrating seven distinct architectures developed to enhance patient detection accuracy by analyzing hospital surveillance images for rapid and precise COVID-19 identification, serving as a crucial first step in automated patient assessment. Various DL-based object detection models, including You Only Look Once (YOLO), Single Shot MultiBox Detector (SSD), Region-Based Convolutional Neural Network (RCNN), and Region-Based Fully Convolutional Network (R-FCN), were evaluated, with results showing that while YOLO and VGG models enable fast detection, the proposed Deep Ensemble of Adaptive Architectures (Ensemble-DL) achieved the highest accuracy, demonstrating its potential for real-world healthcare implementation. Beyond detection, integrating deep learning with soft robotics presents a transformative approach to patient assessment by allowing automated systems to perform essential diagnostic tests, utilizing a soft robot to conduct basic assessments on detected patients, streamlining preliminary medical evaluations, and alleviating the burden on nursing staff. Through extensive real world testing, the Ensemble-DL model outperformed baseline approaches, including Faster-RCNN, R-FCN variants, and CNN+LSTM models, achieving an impressive accuracy of 98.32%, underscoring the growing role of AI-driven solutions in modern healthcare. By enabling precise patient classification and automated assessments, deep learning-based nursing soft robots offer a scalable solution to enhance healthcare efficiency, optimize resource allocation, and improve patient outcomes in both pandemic and post-pandemic scenarios [79].

Advanced imaging analysis techniques, particularly multimodal imaging and deep learning-based segmentation, hold significant potential for studying COVID-19’s impact on various organs by integrating modalities, such as CT, ultrasound, MRI, and molecular imaging (PET/SPECT) to provide a comprehensive assessment of virus-induced tissue damage. Similar to how different MRI modalities combined in brain tumor segmentation, this approach enables the identification of lung inflammation, cardiac fibrosis, vascular abnormalities, and metabolic disruptions, offering complementary insights into disease progression. DL models, which have proven effective in segmenting brain tumors, could be adapted to delineate and quantify COVID-19-induced organ damage, including pulmonary involvement, myocardial injury, and vascular changes, facilitating precise, objective quantification of disease effects for early detection of complications, personalized treatment monitoring, and deeper insights into COVID-19 mechanisms [80].

The integration of robotics and artificial intelligence in pandemic response, particularly in COVID-19, has demonstrated significant potential in enhancing healthcare efficiency, optimizing resource allocation, and improving patient outcomes. AI-driven deep learning models, combined with robotic systems, enable rapid patient detection, automated assessments, and precise classification. Furthermore, understanding the interaction between COVID-19, RAS, and ACE2 expression can refine AI models for risk stratification and personalized treatment strategies. As technology advances, the fusion of AI, robotics, and biomedical research will continue to play a crucial role in managing infectious diseases.

## 8 ANG-(1-9) AND COVID-19

Limited research exists on Ang-(1-9) in COVID-19. Most knowledge centers on ACE2 and Ang-(1-7). Ang-(1-9) is produced by ACE2 like Ang-(1-7). Exploring Ang-(1-9)'s role in COVID-19 warrants consideration.

### 8.1 Clinical Trials Currently Underway

Only six completed studies with published results have explored the therapeutic potential of the components of counter-regulatory RAS in the treatment of COVID-19 (Table **1**). However, multiple ongoing studies aim to deepen our understanding of the COVID-19 receptor-RAS interaction, with examples including: “Angiotensin 1-7 as a Therapy for Pneumonia Caused by Coronavirus 2 (SARS-CoV-2)” (NCT04605887): A phase 2, double-blind, randomized study focuses on Ang 1-7 investigates subcutaneous Ang 1-7 therapy at 500 mcg/kg/day to address the potential role of ACE-2-Ang-(1-7)-MasR in mitigating SARS-CoV-2's effects. “Evaluation of the Possible Role of Angiotensin Peptide (1-7) on Treatment of COVID-19” (NCT04375124): assesses the impact of Angiotensin Peptide (1-7) supplementation using Ang-(1-7) derived plasma on COVID-19 treatment, with a focus on potential Ang-(1-7) deficiency and hyperinflammation. The “Randomized Clinical Trial Phase I/II for the Use of Angiotensin-(1-7) in the Treatment of Severe Infection by SARS-CoV-2” (NCT04633772): A controlled clinical trial evaluates intravenous Ang-(1-7) infusion in severe cases. Effect of Angiotensin-Converting Enzyme Inhibitor and Angiotensin Receptor Blocker Initiation on Organ Support–Free Days in Patients Hospitalized with COVID-19 (NCT02735707): a randomized clinical trial that studies whether ACE inhibitor or angiotensin receptor blocker (ARB) initiation improves outcomes in hospitalized COVID-19 patients. Evaluation of the Potential Benefit of Renin-angiotensin System Inhibitors (RASi, ACEi/ARB) in High-risk Patients with COVID-19. The COVID-RASi Trial (NCT04-591210): An international randomized clinical trial aimed at assessing the potential benefits of angiotensin modulators (Angiotensin converting enzyme inhibitor and Angiotensin II Receptor Blockers) on clinical outcomes in COVID-19 patients. These studies provide insights into the complex role of the renin-angiotensin system in COVID-19 and the potential therapeutic implications of modulating this system. However, the outcomes vary, indicating the need for further research to fully understand the benefits and risks associated with these interventions. Table **1** presents a concise overview of completed studies focusing on the assessment of renin-angiotensin system components and their relevance in COVID-19.

## CONCLUSION

The emergence of SARS-CoV-2 and the subsequent COVID-19 pandemic have spurred extensive research into the relationship between the renin-angiotensin system (RAS) and COVID-19.

ACE2, a key component of the RAS, is closely linked to SARS-CoV-2 infection and has a significant impact on the body's response to the disease. Studies suggest that ACE2 and its product, Ang-(1-7), may influence disease severity and susceptibility, with factors like age, gender, and comorbidities playing crucial roles. Ang-(1-7) therapy is considered a potential approach to mitigate the virus's cardiorespiratory effects, but the intricate nature of this relationship necessitates further research for a comprehensive understanding and practical application in clinical settings. In conclusion, the relationship between the classical and counter-regulatory RAS pathways in COVID-19 is an area of active research that holds significant promise. Understanding the roles of ACE2, Ang-(1-7), and potentially Ang-(1-9) in infection susceptibility and severity may lead to more effective and personalized therapeutic strategies. As we await the results of ongoing clinical trials, it is evident that the RAS system is a critical player in the battle against COVID-19, and its potential for intervention to prevent severe outcomes and long-term complications is an exciting prospect in the field of medicine.

## Figures and Tables

**Table 1:** Completed clinical trials investigating the RAS-COVID-19 relationship.

**ID**	**Year**	**N**	**Treatment**	**Status**	**Results**
NCT04401423	2022	112	TXA127 0.5 mg/kg per day/ Placebo 0.5 mg/kg per day	Completed	The administration of TXA-127 was found to be safe in patients with severe COVID-19 infection.
NCT04335136	2021	185	RhACE2 APN01 / Physiological saline solution	Completed	Modulating the density of ACE2 on the cell surface by regulating ubiquitination and DUBs shows promise as a viable strategy to diminish the entry of SARS-CoV-2 into cells. Consequently, this approach could hold therapeutic potential not only for treating COVID-19 but also for addressing other lung diseases characterized by inflammation and infection.
NCT04472728	2021	360	BIO101	Completed	BIO101 could offer a new therapeutic option by improving respiratory function and promoting survival in patients who develop severe forms of COVID-19.
NCT04419610	2023	30	TRV027/ sodium chloride 0.9%	Completed	Exposure to TRV027 was linked to a tendency towards decreasing D-dimer levels, which did not reach statistical significance.
NCT04924660	2023	343	0.5-mg/kg intravenous infusion of TXA-127 once daily for 5 days or placebo / A 12-mg/h continuous intravenous infusion of TRV-027 for 5 days or placebo.	Completed	Pharmacological interventions that increase angiotensin (1-7) don´t improve outcomes for patients with severe COVID-19.
NCT05615792	2023	11,231	ARB / non-ARB	Completed	ARB use was associated with lower all-cause mortality in COVID-19 patients with cardiovascular diseases.

## LIST OF ABBREVIATIONS

ACE: Angiotensin-converting Enzyme
ACE2: Angiotensin Conversion Enzyme Type 2.
ACEIs: Angiotensin-converting Enzyme Inhibitors
AHT: Arterial Hypertension
Ang II: Angiotensin II
Ang-(1-7): Angiotensin-(1-7)
Ang-(1-9): Angiotensin-(1-9)
ARBs: Angiotensin II Receptor Blockers
AT1R: Angiotensin II type 1
AT2R: Angiotensin II type 2
COVID-19: Coronavirus Disease 2019
CT: Computed Tomography
HE: Hemagglutinin Esterase
IBD: Inflammatory Bowel Disease
ICU: Intensive Care Unit
JAK: Janus Kinase
MasR: Mas Receptor
MRI: Magnetic Resonance Imaging
NEP: Neprilysin
PET: Positron Emission Tomography
R-FCN: Region-Based Fully Convolutional Network
RAS: Renin-angiotensin System
RCNN: Region-Based Convolutional Neural Network
SARS-CoV-2: Severe Acute Respiratory Syndrome
SSD: Single Shot MultiBox Detector
TMPRSS-2: Transmembrane Protease Serine Protease-2
YOLO: You Only Look Once
